# Changes in Optical Properties upon Dye–Clay Interaction: Experimental Evaluation and Applications

**DOI:** 10.3390/nano11010197

**Published:** 2021-01-14

**Authors:** Giorgia Giovannini, René M. Rossi, Luciano F. Boesel

**Affiliations:** Empa, Swiss Federal Laboratories for Materials Science and Technology, Laboratory for Biomimetic Membranes and Textiles, Lerchenfeldstrasse 5, CH-9014 St. Gallen, Switzerland; giorgia.giovannini@empa.ch (G.G.); rene.rossi@empa.ch (R.M.R.)

**Keywords:** clay particles, montmorillonite, spectroscopy, fluorescence, fluorescent enhancement, applications

## Abstract

The development of hybrid materials with unique optical properties has been a challenge for the creation of high-performance composites. The improved photophysical and photochemical properties observed when fluorophores interact with clay minerals, as well as the accessibility and easy handling of such natural materials, make these nanocomposites attractive for designing novel optical hybrid materials. Here, we present a method of promoting this interaction by conjugating dyes with chitosan. The fluorescent properties of conjugated dye–montmorillonite (MMT) hybrids were similar to those of free dye–MMT hybrids. Moreover, we analyzed the relationship between the changes in optical properties of the dye interacting with clay and its structure and defined the physical and chemical mechanisms that take place upon dye–MMT interactions leading to the optical changes. Conjugation to chitosan additionally ensures stable adsorption on clay nanoplatelets due to the strong electrostatic interaction between chitosan and clay. This work thus provides a method to facilitate the design of solid-state hybrid nanomaterials relevant for potential applications in bioimaging, sensing and optical purposes.

## 1. Introduction

Montmorillonites (MMTs) belong to the smectite group of clay minerals. They are composed of repetitions of two tetrahedral sheets (T; Si-O_4_) sharing oxygens with single octahedral sheets (O; Al-O_6_) and are therefore classified as 2:1 clay (T:O ratio; T-O-T). MMTs differ from other members of the smectite group (i.e., beidellite, nontronite) by a few important characteristics besides the different chemical composition (Formula [(Na,Ca)_0.33_(Mg,Al)_2_(Si_4_O_10_)(OH)_2_nH_2_O]): (i) the high surface–volume ratio of the plate-shaped MMT particles, with a thickness and diameter of 1 nm and 0.2–2 μm, respectively; (ii) the surface of MMT particles is slightly negatively charged due to the predominance of oxide anions in the interface, and this charge increases if isomorphic substitutions of Si^+4^ with Al^+3^ and Al^+3^ with Mg^+2^ take place. Most importantly, MMTs are characterized by (iii) the highest hydration extent among clay minerals, which determines their high cation exchange capacity (CEC) (80–150 meq/100 g), mainly Mg^+2^, Ca^+2^, Fe^+2^, Na^+^ and H^+^ [1]. When absorbing water, the space between the layers can remarkably increase, reaching delamination or complete dissociation of the layers (exfoliation) [2,3]. This hydration capacity and CEC have made MMTs very interesting materials for the development of drug delivery systems [4,5] and the preparation of composite materials with novel chemical, physical and mechanical properties [6,7,8,9].

Due to the changes in photochemical and photophysical properties of the dye once it interacts with clay, many researchers have focused their efforts on developing new optical hybrid materials with unique properties. Such composites can find applications in different research areas such as sensing [10] and photocatalysis [11] and have been exploited to improve the thermal [12], photochemical [13] and pH stability [14,15] of the incorporated dye [16]. Furthermore, different methods have been explored in order to increase the adsorption capacity of MMT particles such as calcination, ionic liquid/acidic treatment [17,18] or surface modification [19,20].

Changes in the optical properties of dyes after interaction with clay is the result of dye–dye and dye–clay direct/indirect interactions, where the degree of such interplay is strongly related to the intrinsic properties of both molecules and minerals. In particular, depending on the chemical structure of the dye, adsorption can be mainly driven by hydrophobic/electrostatic interactions or hydrogen bonding. Moreover, even experimental parameters such as dye concentration, pH and temperature can determine the nature, kinetics and degree of adsorption [21]. Among others, ion exchange and the partial negative surface charge have been considered the primary mechanisms of dye adsorption on MMT. It should be noted that the surface charge density of clay strongly determines how the dye will interact with the particles in terms of distribution and orientation on the surface [22,23].

From a theoretical point of view, MMT presents essentially two sites of contact with organic molecules: (1) the surface of the layers presenting siloxane sites, basal interaction; and (2) the edge of the aluminosilicate sheets, interlayer interaction. The basal interactions are determined by the reactivity of the charge distribution on the silica layer in the hexagonal cavity formed by six tetrahedra Si sharing/bordered by six O and an OH group. Molecules presenting -COOH functional groups can form H-bonds with the O atoms at the border of the siloxane hexagonal ring. The negative charge of the surface allows a dipole–dipole interaction with the positively charged alkane hydrogens and direct electrostatic interactions. Interlayer interactions represent an additional site of interplay with polar molecules due to the exchangeable cations present in the interlayer. Some of these cations can increase the negative charge of the oxygen atoms in the silica layers indirectly favoring electrostatic interactions, whereas some monovalent cations such as Na^+^ can directly induce ion–dipole attractive forces with negatively charged molecules. Moreover, the OH groups present at the edge of the MMT sheets favor the formation of H-bonding and dipole–dipole forces at this site of the particles [24].

Theoretical models and molecular dynamics simulations have been used to explore the interplay of organic molecules and clay minerals at the molecular level. Marry et al. showed that hydrogen atoms are attracted in closer proximity to the silicate layer than oxygen atoms [25]. Katti et al. studied, in particular, the formation of noncovalent interactions, i.e., van der Waals and electrostatic forces, between organic molecules with long-backbone chains and MMT. They observed that electrostatic forces mainly drive the interplay between organic molecules and clay. In particular, the higher the positive charge of the atoms forming the functional groups, the higher is the interaction with the clay; on the contrary, a partial negative charge determines repulsions between the organic molecules and clay. According to their simulations, H and C atoms lead to attractive forces with clay, whereas other atoms (O and N) ensure repulsive forces (Table S1) [26]. However, as simulated by Heinz et al., once the NH_2_ group is protonated (-NH_3_^+^), the situation changes, and repulsions become attractive forces instead, confirming the importance of environmental parameters such as pH for the molecule–clay interplay. H atoms can form H-bonds with O atoms in the silicate surface (N-H…O-Si) besides the electrostatic (ionic–dipole) interaction that occurs between the cationic NH_3_^+^ group and the negatively charged MMT cavities [27].

Chitosan, a linear polysaccharide composed of β-(1-4)-linked D-glucosamine, is an excellent choice to exploit these findings. At a slightly acidic pH (~5.5), the amino groups are ionized, allowing a strong interaction with clay. Accordingly, this property has been exploited in the design of MMT–chitosan nanocomposites, where the polymer chains are strongly adherent to the MMT platelets [28,29,30]. Under adequate conditions, the chitosan chains were fully extended due to the regular attachment of the protonated amino groups. Such behavior constitutes an excellent platform to facilitate the interaction of dyes (covalently attached to the chitosan chains) and MMT platelets.

In the present work, we evaluated the changes in the optical properties of different dye–chitosan conjugates after interaction with MMT at different concentrations (10, 1 and 0.1 wt.% of MMT in DI water). The synthesized fluorescent polymers were tested, and the changes in the optical properties of the fluorescent polymers were evaluated. The results were compared with those achieved using nonconjugated dyes, confirming that the optical behavior of fluorophores is not altered once covalently linked to chitosan. On the contrary, we showed that the well-known self-quenching mechanism of fluorescein isothiocyanate (FITC) can be avoided by exploiting the stretching capability of chitosan on the MMT surface. Aiming to explain which molecular features are responsible for the adsorption of the molecules on the mineral and changes in the optical behavior after dye–MMT interaction, we evaluated our experimental data using known molecular dynamics methods. The findings presented herein represent a useful tool in the process of selecting the proper dye for the development of new hybrid materials. Furthermore, we evaluated the properties of hybrid materials in detail, combining a common dye such as FITC with MMT, and considering the remarkable fluorescence enhancement achieved, we proposed novel applications convenient for different scientific communities.

## 2. Materials and Methods

### 2.1. Materials

Deacetylated chitosan (medium viscosity), fluorescein isothiocyanate (FITC), fluorescein (Fluo), rhodamine isothiocyanate (Rho), eosin isothiocyanate (E), 5(6)-carboxynaphthofluorescein (CNF), 5(6)-carboxy-2′,7′-dichlorofluorescein (CCF), 7-amino-4-methyl-3-coumarinylacetic acid (CNH2), 7-hydroxy-4-methylcoumarin (COH), glycerol, Tween20, and 1–4 dioxane were all purchased from Sigma-Aldrich (Buchs, SG, Switzerland). Montmorillonite Dellite^®^ LVF (CEC: 105 meq/100 g) was donated by Laviosa Chimica Materia (Livorno, Italy). Fluorescamine was purchased from TCI (TCI Germany GmbH, Eschborn, Germany). Phosphate-buffered saline (PBS) (10 mM) was prepared by solubilizing NaCl (0.137 M), KCl (2.7 mM), Na_2_PO_4_ (0.01 M) and KH_2_PO_4_ (1.8 mM) in DI water. The pH was adjusted by adding NaOH and HCl.

### 2.2. Methods

**Scheme 1:**
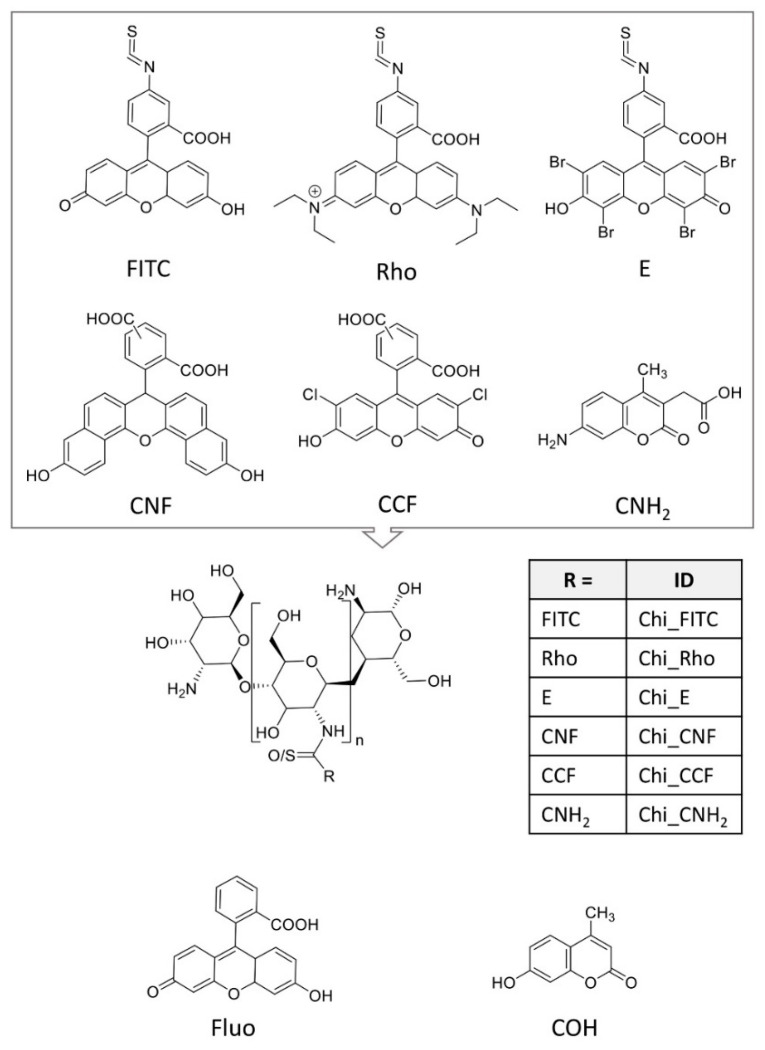
Structure of dyes tested in the present study. Fluorescein isothiocyanate (FITC), rhodamine isothiocyanate (Rho), eosin (E), 5(6)-carboxynaphthofluorescein (CNF), 5(6)-carboxy-2′,7′-dichlorofluorescein (CCF) and fluorescein (Fluo) belong to the xanthene family, and 7-amino-4-methyl-3-coumarinylacetic acid (CNH_2_) and 7-hydroxy-4-methylcoumarin (COH) are coumarin derivatives. Six of these tested dyes were conjugated with chitosan. The reached fluorescent polymers were then used in the experiments (Chi_FITC, Chi_Rho, Chi_E, Chi_CNF, Chi_CCF, and Chi_CNH_2_).

*Chitosan-conjugation protocol:* Deacetylated chitosan (5 mg) was stirred in 1 mL of 1M HCl overnight. The sample was diluted up to 4 mL, and the sample was neutralized to pH 6 using a solution of 1M of NaOH. After 5 h of stirring, the solution was filtered through cotton wool, and the dye (i.e., FITC, Rho, E, CCF, CNF or CNH_2_) was added, reaching 1.1 mM as the concentration in the final solution (5 mL). The concentration of dye was designed to have one molecule of dye for every five units of glucosamine in the chitosan chain. The reaction was stirred in the dark for 24 h. The unreacted dye was removed by 24 h of dialysis (cut-off membrane of 14 kDa), followed by cycles of centrifugation–redispersion in DI water (3×, 10 min, 8000 rpm). After purification, the volume was adjusted with DI water to 5 mL (1 mg/mL of chitosan), and the concentration of dye was determined by measuring the absorbance signal and using the calibration curve plotted for each dye. A scheme of the conjugation procedure can be found in the ESI (Figure S1). The same procedure was used for the preparation of chitosan conjugated with different concentrations of FITC, Chi5:1_FITC, Chi5:2.5_FITC and Chi5:5_FITC but using the ratios between the units of glucosamine and FITC of 5:1, 5:2.5 and 5:5, respectively (Scheme 1). The amount of dye quantified using a calibration curve and 0.24, 1.29 and 3.52 mM was the calculated amount of FITC per 1 mg/mL of, respectively, Chi5:1_FITC, Chi5:2.5_FITC and Chi5:5_FITC.

*Concentrations of dye used in the experiments:* The concentration of the free and chitosan-conjugated dyes used for all experiments was chosen after evaluating the absorbance and fluorescent signals at different concentrations. Samples were used at the same concentrations in all experiments. These concentrations were chosen to have absorbance signals below 1 A.U. and measurable fluorescent signals, avoiding self-quenching. They were 1 μM for FITC, Chi_FITC, Fluo, E, Chi_E, CNF, Chi_CNF, CNH_2_ and Chi_CNH_2_ and 0.1 μM for Rho, Chi_Rho, CCF, Chi_CCF and COH. If not differently specified, the concentration indicated for the chitosan-conjugated compounds is intended as the amount of dye conjugated. For each dye, the excitation wavelength was chosen as the one corresponding to the maximum peak of the absorbance spectrum. If not specified, the fluorescent intensity is intended to be the value corresponding to the maximum of the peak of the fluorescent spectra. In particular, the following wavelengths were used for λ_ex_–λ_em_, respectively, for each dye: FITC/Chi_FITC, 490–520 nm; Fluo, 490–515 nm; Rho/Chi_Rho, 550–590 nm; CCF/Chi_CCF, 500/525 nm; E/Chi_E, 520–550 nm; CNF, 390–530 nm or 550–660 nm; CNH_2_/Chi_CNH_2_, 340–450 nm; COH, 320–460 nm or 370–460 nm.

*MMT samples’ preparation:* MMT particles were suspended in DI water at 10, 1 and 0.1 wt.% concentrations. The morphology and composition of MMT used in this study are reported in the ESI (Figure S9) (SEM, XRD, EDX). For brevity, we named these samples 10C, 1C, and 0.1C, respectively.

In the experiments in Section 3.1.1 and Section 3.1.2, the defined amount of dyes was treated with MMT samples 10C, 1C and 0.1C, as specified, and after 15 min, the fluorescent signal was measured once excited at the specific excitation wavelength.

In the experiments of Section 3.1.1 and Section 3.1.2, dye and MMT were mixed using 100 µL of the stock solution of the dye (reaching the above-mentioned concentrations) and 100 µL of the 1 wt.% MMT sample, reaching 200 µL as the final volume.

In the experiment discussed in Section 3.1.3, 100 µL of dye was mixed, reaching the above-mentioned concentrations with 100 µL of the specific solvent evaluated (DI water, 1 wt.% MMT, 2% agarose, glycerol, Tween20 and 1–4dioxane).

In the experiments in Section 3.2 For the *pH* study, dye samples were prepared by diluting the Chi_FITC stock solution using PBS at different pH values (from 3 to 9). A 100 µL volume of these solutions was mixed with 100 µL of DI water or MMT at different concentrations (10 and 0.1 wt.%). The concentration of Chi_FITC and FITC in the final volume (200 µL) was 0.1 µM. For the environment study, 50 µL FITC or Chi_FITC was mixed with 100 µL of the different solvents tested in the study (water, 2% agarose, glycerol, Tween20), and a further 50 µL of water or MMT dispersions at 10% or 1 wt.% was added. The final concentration of FITC and Chi_FITC was 1 µM. Alternatively, 100 µL of FITC or Chi_FITC was mixed with glycerol, reaching 1 µM as the final concentration of the dye and 50, 25, 10, 5 and 0 wt.% of glycerol in the final volume of 200 µL.

For each experiment, 200 µL of the samples described above was added in a 96-well plate, and after 15 min, the spectra were recorded using a UV–vis spectrophotometer (Varian 50Bio connected to 50 MPR, Agilent, Santa Clara, CA, US) and a fluorescent spectrophotometer (Cary Eclipse, Agilent, Santa Clara, CA, US). All spectra were normalized with the spectrum acquired for the corresponding solvent (i.e., water, MMT, 2% agarose, Tween20, etc.),

For confocal laser scanning microscope analysis (CLSM), FITC and Chi_FITC at the concentration of 0.1 µM were treated with 1 wt.% MMT, and 5 µL of these dispersions were dropped on microscope slides. Images were acquired using a 490 nm LASOS Ar-lon Laser (Model LGN 3001, Remote Control RMC 7812 Z2, Zeiss, Jena, Germany) in a dark incubation chamber of the laser scanning confocal microscope (LSM 780, Zeiss, Jena, Germany). Images were analyzed using the software ZEN 3.1 blue edition (Zeiss, Jena, Germany).

## 3. Results and Discussion

### 3.1. Dye–MMT Interaction: Experimental Analysis of Optical Properties

We analyzed the relationship between the structure of organic molecules and their adsorption on MMT particles and identified key aspects of the molecular structure, which could allow predicting changes in the optical properties upon dye–clay interaction. In particular, we tested six dyes belonging to the xanthene family (FITC, Rho, E, CNF, CFF and Fluo) and two coumarin derivatives (CNH_2_ and COH), which are commonly used for biological and nonbiological applications due to their good fluorescent properties and biocompatibility (Scheme 1, Section 2.2). In order to promote adsorption and avoid the well-documented tendency to form molecular aggregates on the particles’ surface [31,32], six of the tested dyes (FITC, Rho, E, CNF, CCF and CNH_2_) were previously conjugated with chitosan, and the achieved fluorescent polymers (Chi_FITC, Chi_Rho, Chi_E, Chi_CNF, Chi_CCF and Chi_CNH_2_) were then adsorbed on the MMT particles (Figure S1).

#### 3.1.1. Absorbance and Fluorescent Spectra

In the first experiment, we chose the intermediate concentration of MMT as a reference, i.e., 1C, to evaluate the optical changes after dye¬–clay interactions. Therefore, both the free dyes and chitosan-conjugated dyes were mixed with 1C or diluted with water, and the UV–vis and fluorescence spectra were recorded after 15 min (Figures S2–S4). Considering that bathochromic shifts (red-shift) of 5–10 nm observed for FITC, Rho, E, CCF and Fluo (Figure S2) are typical of the dye transition from solution into a solid matrix, we can confirm that most of the dyes were effectively adsorbed on the MMT surface [33]. Besides the increase in the intensity signal of FITC, only small variations were observed in the absorbance spectra (Figure S2). Whereas a remarkable enhancement of the signal in the fluorescent spectrum was measured for FITC and CCF (intensity ca. fivefold higher) for Fluo, Rho and E, the enhancement was negligible (c.a. 1.3 times higher). The difference between FITC and Fluo is ascribed to the expected lower adsorption of the latter on the negatively charged MMT due to the presence of the COOH group. A bathochromic shift in the absorbance spectrum was observed instead for CNH_2_ when treated with MMT (from 340 nm to 360 nm), and the fluorescent signal measured at 450 nm was found to be around three times higher than that measured for CNH_2_ in water.

The difference between FITC, E and CCF lies in the presence of halogens (E-Br; CCF-Cl). The large steric hindrance of Br atoms surrounding the OH, usually involved in the MMT interaction [25], present in E could explain the absence of changes in the absorbance and fluorescent spectra [34]. The low-affinity of rhodamine-like dye for clay is well documented due to its tendency to exist mainly as a dimer [35]; however, the small red-shift (10 nm approximately) and the small enhancement in the measured fluorescent signal can indicate that it indirectly interacts with the MMT surface and it resides in the double layer [36].

The bathochromic shift observed for CNH_2_ when treated with MMT indicates the good dye–clay interplay of the primary amine after protonation [27]. For the 7-amino derivate coumarin, changes in optical properties have been explained by the planar intramolecular charge-transfer (ICT), which allows the resonance between the amino group and the benzopyrene moieties [37,38].

**Figure 1:**
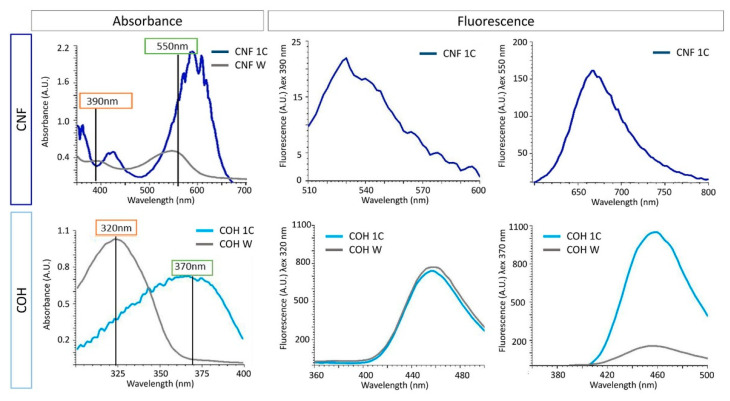
Absorbance and fluorescent spectra of CNF and COH. The remarkable changes in the optical properties between such dyes in water and in the presence of montmorillonite (MMT) are due to intermolecular electronic transitions such as π–π* (COH) and n–π* (CNF). All spectra were normalized to the signals of the corresponding reference (water (W) or MMT suspension).

The results of CNF and COH are noteworthy. In these two cases, we observed the largest variations in the optical properties of the dyes when treated with MMT, as shown in Figure 1. In the case of CNF, the main peak in the absorbance spectrum was red-shifted to 600 nm in the presence of MMT, and the intensity was three times higher. The changes in the absorption spectra of CNF could find an explanation in the photoactivity theory, which relates the bathochromic shift in the absorbance peak to the reduced affinity of the proton for the molecule in the excited state and the stabilization of the acid form [39]. In this approach, the aromatic ring becomes more electronegative in the excited state, shifting some electron density away from the oxygen, making it a weak base (n–π* transition). It has been proven that, in water, excited-state proton transfer (ESPT) may occur via the formation of a water bridge between a proton-donating site (OH) and a proton-accepting site on the photoacid molecule [40,41]. In our specific case, the bathochromic shift of the CNF absorbance from 550 nm (in water) to 600 nm (with MMT) could be assigned to the formation of H-bonds between the weakened molecular OH and the OH at the edge of the MMT particles. When excited at both excitation wavelengths (i.e., 390 and 550 nm), only the samples treated with MMT showed a relevant fluorescent signal. Similarly, the maximum absorbance peak of COH was red-shifted from 320 nm to 370 nm when treated with MMT. The bathochromic shift observed for COH has been explained as the result of electronic transitions π–π*, which are related to the charge transfer from the benzoic cycle and the pyranone moiety [42]. However, this π-interaction can occur according to the charge-transfer theory, even between the lone pair electrons of the surface oxygen in the silica layer and the dye molecule [23]. When excited at 370 nm, the fluorescent signal measured at 460 nm was 10 times higher for COH treated with MMT compared with the one in water. Interestingly, the red-shift observed for COH from 320 to 370 nm was only completed by using higher concentrations of MMT, as shown in Figure S3. This proved that it is effectively the dye–clay interaction that determines the change in the optical properties, excluding other phenomena such as dye aggregation.

Figure S4 shows the absorption and fluorescent spectra of the chitosan-conjugated dye in the solution treated with 1C MTT. Considering that no shifts were observed in the absorbance and fluorescence spectra of, respectively, free and conjugated dyes, the interactions with MMT employ similar mechanisms. However, the fluorescent enhancement observed in the case of Chi_Rho, which is not observed using Rho, suggests that the conjugation with chitosan favors the interaction with clay.

#### 3.1.2. Fluorescent Enhancement of Free and Conjugated Dye–MMT Hybrids

**Figure 2:**
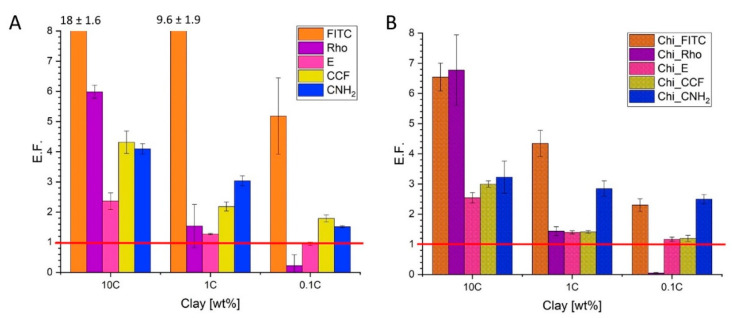
Enhancement factor (E.F.) calculated for nonconjugated dyes (**A**) and chitosan-conjugated dyes (**B**). E.F. was calculated by dividing the fluorescent intensity measured for the sample treated with MMT at different concentrations (10C, 1C and 0.1C) by the intensity measured for the dye in water at the maximum of the emission peak. Mean ± SD (*n* = 3). The intensity was determined using the following wavelengths as λ_ex_–λ_em_: FITC/Chi_FITC, 490–520 nm; Rho/Chi_Rho, 550–590 nm; CCF/Chi_CCF, 500/525 nm; E/Chi_E, 520–550 nm; CNH_2_/Chi_CNH_2_, 340–450 nm.

For each free and chitosan-conjugated dye, we calculated the fluorescent enhancement factor (E.F.) as the ratio between the fluorescence intensity measured for the molecule treated with MMT at different concentrations and the signal measured in water. The values are reported in Table S2 in the ESI. This experiment was not conducted for CNF and COH due to the relevant bathochromic shift in the absorbance peak when treated with MMT. Figure 2 shows the E.F. calculated for the nonconjugated dyes (Figure 2A) and chitosan-conjugated dyes (Figure 2B) treated with 10C, 1C and 0.1C. Outstanding results were observed after treating FITC with MMT at any concentration, and a slightly lower enhancement was measured for Chi_FITC. As noticed, in particular with FITC, Chi_FITC, Rho and Chi_Rho, the higher the concentration of the MMT particles, the higher the E.F. Only in the case of Rho and Chi_Rho the E.F. was a result <1 calculated when treated with low concentrations of MMT (0.1C), which could indicate that, in such conditions where the ratio Rho/MMT is unfavorable for the adsorption of the dye on MMT, fluorescence is quenched rather than enhanced, likely due to aggregation of the dye in the solution.

**Figure 3:**
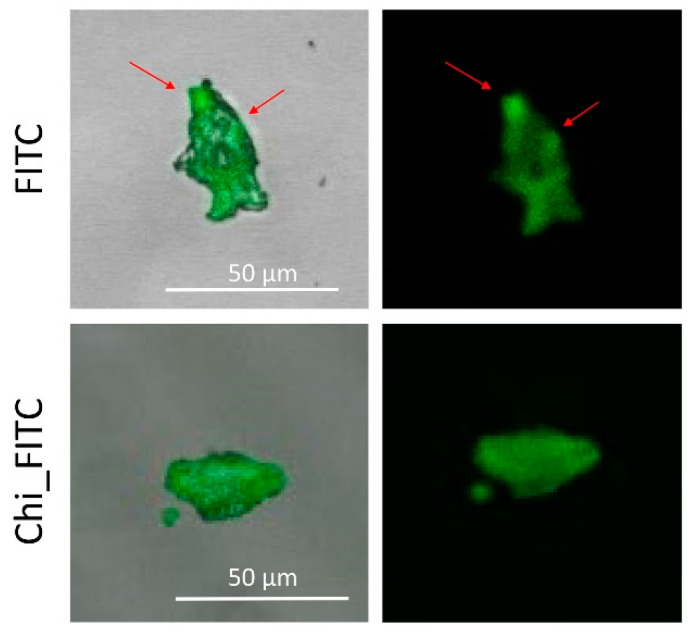
Confocal laser scanning microscopy (CLSM) analysis of FITC and Chi_FITC treated with 1C MMT.

A few differences were noticed when evaluating the E.F. values calculated for the nonconjugated and conjugated dyes. An increase of a factor of one was observed for Chi_Rho treated with 10C and for Chi_NH_2_ treated with 10C and 0.1C compared to the free dye. This could indicate that the interaction of such dyes with clay is favored once conjugated with chitosan. On the other hand, in the case of FITC and CCF, the situation is the opposite. This could find an explanation in the stabilization effect achieved when conjugating the dye to the polymer. It is well known that the formation of molecular aggregates (supermolecular assemblies) often leads to dramatic changes in the photophysical properties. According to the so-called “aggregation theory,” even at low concentrations of dye as those tested here, due to the charge distribution at the mineral surface, the organic molecules can form islands of different types of aggregates at the surface rather than being homogeneously distributed. Interactions that may lead to the formation of aggregates on mineral surfaces range from van der Waals, electrostatic forces, hydrophobic effects and hydrogen bonding. Two of the main types of aggregate dye monomers that may form are J-aggregates (head-to-tail) and H-aggregates (sandwich type), and the analysis of the spectra is the best method for probing how the molecules interact. In this regard, the high E.F. measured for FITC could be related to the formation of J-aggregates on the MMT particle’s surface, considering that it has been largely proven that J-aggregates can dramatically increase the fluorescence quantum yield or the nonlinear optical properties [43]. Once conjugated with chitosan (Chi_FITC), the molecules are no longer free to interact with each other, forming aggregates and, therefore, the measured E.F. is smaller. To support this hypothesis, FITC and Chi_FITC treated with 1C MMT were analyzed by confocal laser scanning microscopy (CLSM). As can be seen in Figure 3, in the FITC sample, some spots at a higher fluorescence intensity can be observed on the MMT surface, which indicates the presence of aggregates of dye, while in the case of Chi_FITC, the fluorescence is more uniformly distributed on the mineral particle.

#### 3.1.3. Fluorescent Enhancement: Evaluation of the Possible Mechanisms Involved

We compared the absorbance and fluorescence spectra measured for the dyes at the same concentration in different environments with that obtained with 1C MMT to identify which phenomena can be related to the optical changes in the dye upon MMT interaction. The mechanisms that lead to the alteration of the optical properties of a fluorophore can be divided into (i) general mechanisms related to the surrounding environment (polarity, refractive index, pH, stiffness, etc.) and (ii) specific effects in which the chemical and physical properties of the dye are altered upon interaction with surrounding molecules (formation of H-bonding, acid–base interactions, charge-transfer) [44,45]. We chose 1-4 dioxane (1-4diox) as nonpolar solvents to be compared with the result achieved in water (W) as a polar solvent. Tween20 (Tween) and glycerol (Gly) were selected as solvents with different viscosities, 0.3 and 1.4 Pa⸱s, respectively, and available OH groups for H-bond formation. Finally, the dyes were incorporated into an agarose-based hydrogel (Agar), the stiffness of which reduces the rotational freedom of the dye.

The results achieved highlight the sensitivity of FITC and Chi_FITC to the surrounding environment (Figure S5A,B). They support the hypothesis proposed earlier that the fluorescence enhancement observed for FITC is related to the formation of J-aggregates at the MMT surface, considering that neither indirect effects (i.e., polarity and viscosity) nor direct effects (OH groups) have been shown to induce enhancement but, on the contrary, the quenching of fluorescence. Considering the results observed with E (Figure S5C), we could hypothesize that (i) the optical properties of such dyes are not affected by the increase in environmental stiffness, although a red-shift of both absorbance and fluorescence can be achieved by increasing the viscosity (Tween and Gly) or using apolar solvents (1-4diox). Furthermore, (ii) remarkable fluorescent enhancement can be achieved using molecules having OH groups suitable for the formation of H-bonding (Tween, E.F. 12). A combination of factors may lead to the fluorescence enhancement observed for Rho (Figure S5D). Indeed, both indirect (i.e., viscosity and polarity) and direct solvent factors (i.e., H-bond formation) seemed to induce enhancement of the fluorescent signal. In particular, the intensity of the signal was enhanced in 1-4diox (E.F. 27) due to the solubility of Rho in apolar solvents and solvent reach in OH groups available for H-bond formation (Tween: E.F. 45; Gly: E.F. 40) [46].

**Figure 4:**
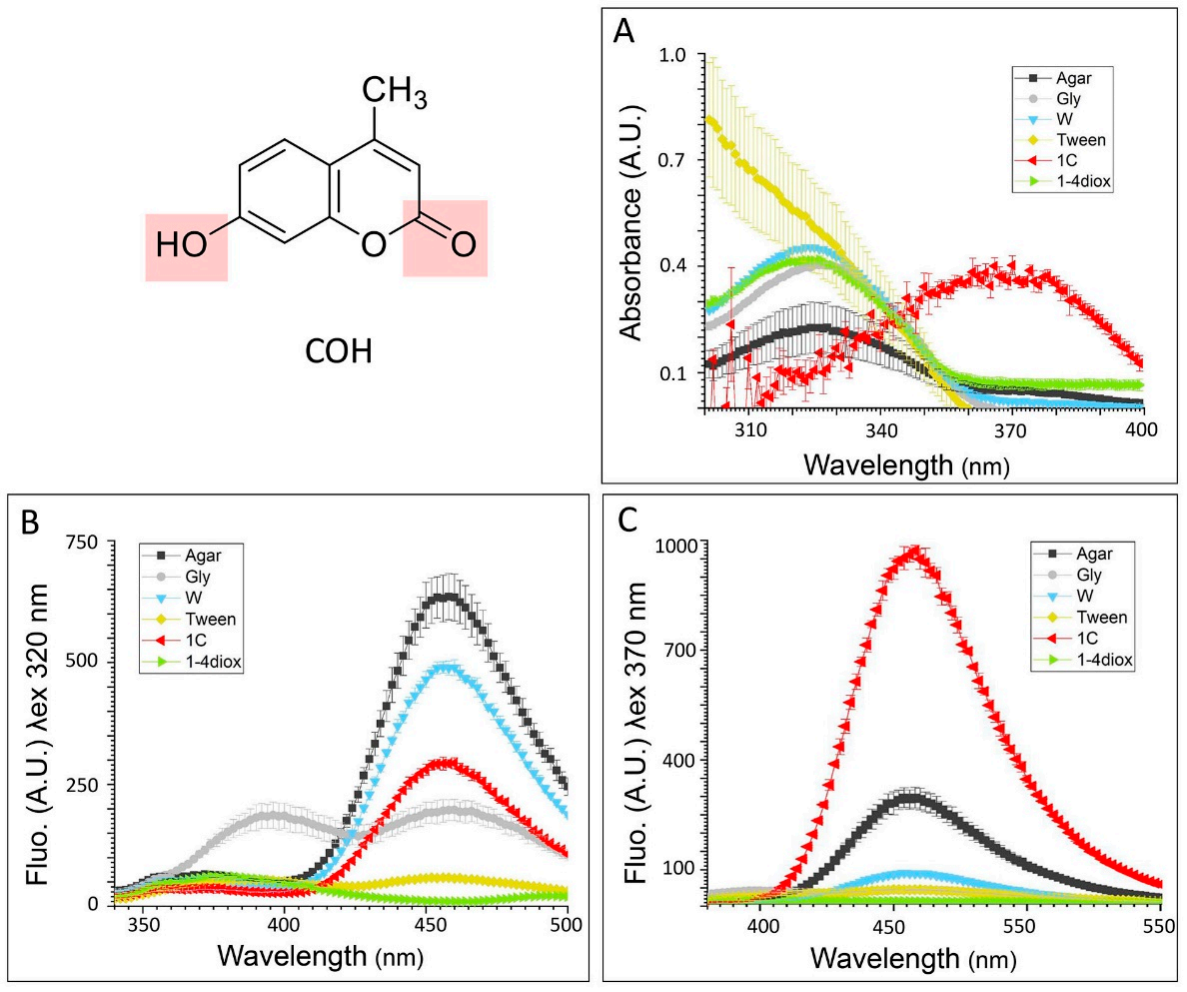
Study of the phenomenon involved in the optical changes of COH. (**A**) Absorbance and (**B**,**C**) fluorescent spectra are reported for each dye dispersed in different solvents: Agar—black line (hydrogel with high stiffness); Gly—gray line (1.4 Pa⸱s; 3 OH groups/molecule; propane-1,2,3-triol); Tween—yellow line (0.3 Pa⸱s; branched polymer with 3 OH group at the end of polyethylene chains; polyoxyethylene sorbitan monolaurate) MMT—red line; 1-4diox—green line (apolar solvent) and water—gray line. All spectra were normalized to the signals of the corresponding solvent and MMT suspension. Data are presented as mean ± SD (*n* = 3). The relatively large SD observed especially for Tween and Agar can be explained by the strong absorbance of the solvent itself around 300–350 nm.

Of particular interest were the results achieved for COH. As shown in Figure 4A, the absorbance peak is only shifted when COH is treated with MMT. This indicates that neither indirect effects (polarity, viscosity) nor direct effects (H-bonding) can be attributed to the optical variations observed when treating COH with MMT, which is the bathochromic shift of the absorbance peak from 320 to 370 nm. Exciting all samples at the usual wavelength of COH (320 nm; Figure 4B), the fluorescent signal measured for the MMT sample is of low intensity, but the peak is not shifted (460 nm). On the other hand, in the Gly, Tween and 1-4diox samples, a new peak at 390 nm was detected, which can be ascribed to a partial molecular destabilization or the formation of H-bonds. Besides the flattening of the molecule at the MMT surface, it has been proposed that dipole–dipole interaction may occur between the fluorophore and MMT leading to the enhancement of the fluorescence signal of the coumarin derivative [47]. A bathochromic shift of both absorbance and fluorescent peaks was observed for CNF only when treated with MMT, which highlights the peculiar effects achievable when combining dyes and mineral clay in forming hybrid materials (Figure S6).

With the above results, we identified two main mechanisms by which MMT induces changes in the optical behavior of fluorophores: (1) the indirect mechanism and (2) the direct mechanism. The first mechanism requires that the molecules involved present a rotational center in the structure or must be prone to a reorganization of the electron distribution after dimer or aggregate formation. In such a situation, MMT serves purely as physical support, as observed for instance with metal–organic frameworks [48] and quartz substrates [49]. In this case, the fluorophore can aggregate on MMT or its molecular rotation is limited once adsorbed on the clay surface, inducing well-known phenomena such as aggregation-induced emission (AIE), surface-fixation-induced emission (S-FIE) [50] and restriction of intermolecular rotation (RIR) [51]. In the direct mechanism, the atoms of which the silica layers are formed (mainly the negatively charged oxygen atoms) are actively involved, inducing chemical/structural alterations of the molecule, altering its optical behavior. We conclude that the electron density of the oxygen in the silica layer can couple with the electron density of planar aromatic groups. This leads to the formation of π–π interactions that are strong enough to induce the reorganization of the electrons inducing spin–orbit coupling (SOC) [52], internal charge transfer (ICT) or twisted internal charge-transfer (TICT) [53], known mechanisms that alter the optical properties of fluorophores.

### 3.2. Proposed Applications of Free and Conjugated Dye–MMT Hybrids

When choosing a fluorophore for imaging, sensing and biological applications, the key aspects usually considered are: (i) excitation and emission range, (ii) stability and (iii) intensity of the signal. Given the results shown previously, the optical changes observed in the dyes when interacting with MMT could allow interesting applications. For instance, the bathochromic shift in the absorbance peak observed for COH, CNH_2_ and CNF could be of particular interest when the dye is used in biological samples [54,55,56]. In such a situation, the red-shift of the signal is useful to reduce the high background noise related to the interferences of albumin and other proteins in the UV range, which limits the use of dyes with high excitation/emission energies. The high fluorescent enhancement observed treating FITC and Chi_FITC with MMT paves the way for several applications, therefore we further investigated this specific dye–clay interplay.

**Figure 5:**
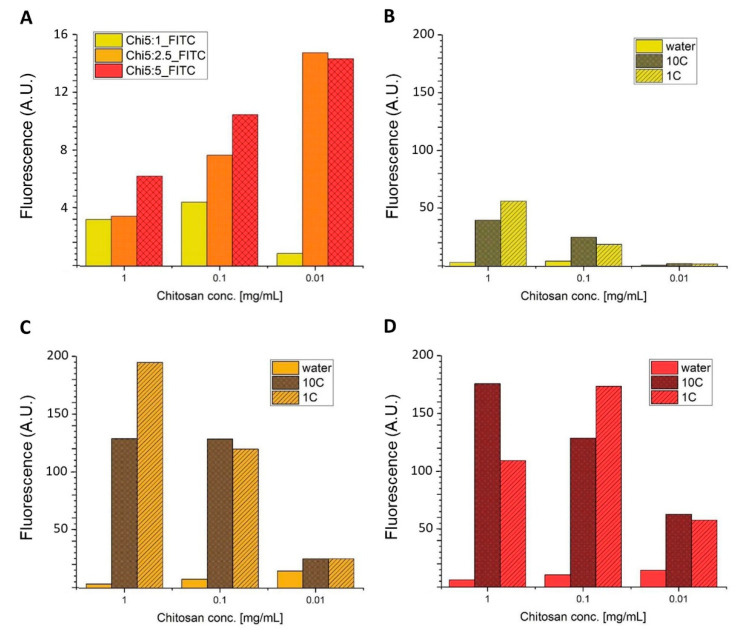
(**A**) Fluorescent signal measured for Chi5:1_FITC, Chi5:2.5_FITC and Chi5:5_FITC in water at different dilutions. Fluorescence enhancement achievable treating Chi5:1_FITC (**B**), Chi5:2.5_FITC (**C**) and Chi5:5_FITC (**D**) with 10C and 1C MMT.

We first exploited the stretching of chitosan upon interaction with the MMT surface to increase the fluorescent signal. For this purpose, we conjugated chitosan with different ratios of FITC, a dye highly sensitive to self-quenching. As noticed from Figure 5A, while for Chi5:1_FITC the signal decreases after dilution, in the case of Chi5:2.5_FITC and Chi5:5_FITC, the fluorescence increases with a decrease in the sample concentration. This indicates that with a high conjugation rate, self-quenching occurs due to the high concentration of fluorophore along the chitosan chains, which are in a random coil conformation in the solution. However, treating the samples with MMT (Figure 5B–D), the fluorescence signal increases dramatically, in particular for Chi5:2.5_FITC (C) and Chi5:5_FITC (D), for which no self-quenching effect was observed as well. As reported in Table 1, remarkable E.F. factors were calculated for the Chi5:2.5_FITC and Chi5:5_FITC samples compared to the E.F. measured for Chi5:1_FITC. This difference is explained by the fact that the self-quenching phenomenon observed for the sample in water is limited once the conjugated polymer chain unfolds and stretches out on the MMT surface. This simple strategy allows for achieving high fluorescent signals without being limited by the self-quenching effect, commonly observed when dyes are used at high concentrations.

One of the properties that have made FITC such a common and widely used dye is its pH responsiveness in a physiological range (pH 5–8). However, as shown for other pH indicators such as methyl red [57], we demonstrated that Chi_FITC, upon interaction with MMT. loses its pH responsiveness. As shown in Figure S7, the fluorescent signal of Chi_FITC increased with the increase in pH, but in the presence of 10C MMT and 0.1C MMT, the pH responsiveness of the dye was completely lost, even at low concentrations, which can be ascribed to the stabilization of the electron distribution of FITC upon interaction with MMT (Figure S7). Such behavior of FITC interacting with MMT will be useful in designing optical hybrid materials for applications where the changes in fluorescence intensity upon pH variation are undesirable, such as in imaging and sensing [58,59].

**Figure 6:**
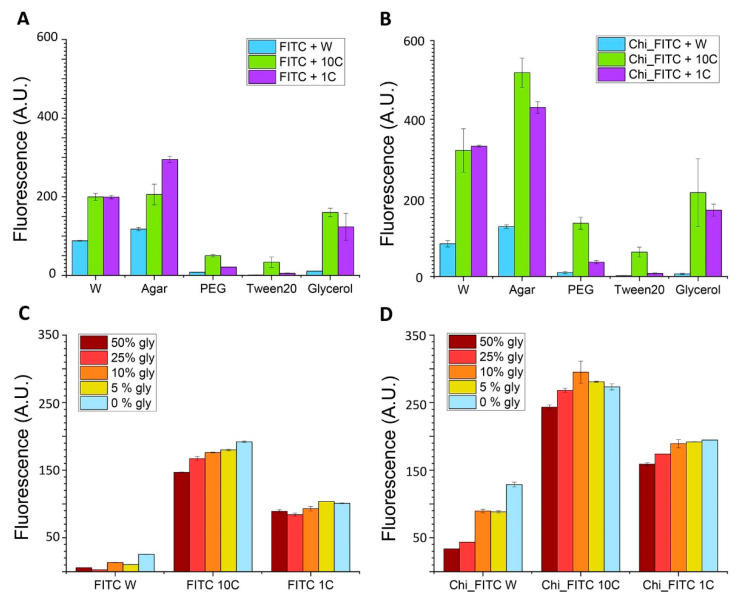
Effect of solvents having different features (agar, PEG, Tween and glycerol) on the fluorescence signal measured for FITC (**A**) and Chi_FITC (**B**). Effect of glycerol at different concentrations on the fluorescence signal of FITC (**C**) and Chi_FITC (**D**).

As previously discussed, the fluorescent properties of dyes vary with the variation of the surrounding environment. Hence, when designing hybrid materials, it becomes of crucial importance to be aware of the different behavior of the fluorophore when its embedding matrix changes even slightly. We measured the fluorescent signal of FITC and Chi_FITC exposed to different environmental conditions. As shown in Figure 6, the fluorescence signal of FITC (Figure 6A) and Chi_FITC (Figure 6B) in 2% agar (hydrogel with high stiffness) is enhanced compared to that in water. On the contrary, in a viscous solvent such as polyethylene glycol (PEG), Tween and glycerol (solvents often used for the formulations of hydrogels, nanomaterials or solid matrices) the fluorescent signal of either FITC and the Chi_FITC is remarkably reduced and hardly distinguishable from the background noise. However, the addition of 10C or 1C MMT can help to overcome such issues, enhancing the fluorescent signal to detectable values. In most of the cases tested, the addition of 10C or 1C MMT leads to an increase of the fluorescent signal with an E.F. ranging from a factor of 2 (FITC and Chi_FITC with Tween and 1C MMT) to a factor of 21 (Chi_FITC with glycerol and 10C MMT). The use of MMT to reduce FITC sensitivity to the surrounding environment may be useful in different fields. For instance, by simply adding MMT to samples composed of FITC/Chi_FITC (Figure 6C,D respectively) with different concentrations of glycerol (50–0%), the fluorescence of the dye can be completely restored or even enhanced. Hence, considering the wide application of glycerin in fields spanning from biology (such as protein storage) to material engineering (such as film fabrication), such a ploy could save researchers’ time and efforts.

Moreover, we exploited the dye–clay interaction for sensing applications. Fluorescamine is a compound commonly used for the “off–on” fluorescent detection of amino acids. As shown in Figure S8A, the molecule itself is not fluorescent, but once it reacts with the amino groups of amino acids, a fluorescent compound is formed, and a signal can be measured at 480 nm (excitation 390 nm). One of the limitations related to the use of fluorescamine is the high background interference and the low fluorescent signal measured in the presence of a low amount of amino acids. As proof of concept, we mixed the fluorescent form of fluorescamine (activated after conjugation with chitosan) with MMT at different concentrations. As shown in Figure S8B, the fluorescent signal measured at 490 nm was enhanced by a factor of 48 in the presence of 10C MMT. Even using 1C of MMT, a remarkable enhancement factor of nine was observed. We believe that such an outstanding fluorescent enhancement will be useful to improve the sensitivity and reduce the limit of detection (LOD) of fluorescent-based sensing approaches.

## 4. Conclusions

In the present work, we evaluated the mechanisms involved in the optical changes of xanthene dyes and coumarin derivatives when in contact with clay nanoplatelets. We observed that key structural parameters can favor the interaction with MMT. Indeed, due to the presence of both OH and NH_2_ in its polymeric chains, chitosan efficiently improved the interaction of dyes with clay particles. We showed that, by tailoring the composition of chitosan-conjugated dye and MMT hybrids, large improvements in the fluorescent properties could be obtained, especially at concentrations well above the self-quenching of the fluorophore. We distinguished between two main mechanisms by which MMT induces changes in the optical behavior of fluorophores: (1) the indirect mechanism, leading to AIE, FIE and RIR phenomena ascribed to the aggregation/adsorption of molecules on the MMT’s surface; (2) the direct mechanism, in which the reorganization of the electron density of the molecule upon interaction with MMT leads to phenomena such as SOC and ICT. Furthermore, we exploited the optical changes achieved after adsorption of the fluorophore on the MMT surface for different applications, i.e., in vivo and in vitro imaging, protein storage, film fabrication and sensing. Combined with the stability provided by the strong electrostatic interactions between chitosan and MMT, our results help in choosing the proper formulation while designing fluorescent hybrid materials carrying specific optical properties.

## Figures and Tables

**Table 1:** E.F. values calculated for Chi5:1_FITC, Chi5:2.5_FITC and Chi5:5_FITC at different dilutions treated with 10C and 1C. Values are calculated as the ratio between the fluorescent value of the samples treated with MMT and in water.

E.F.	Chi5:1_FITC		Chi5:2.5_FITC		Chi5:5_FITC	
mg/mL	10C	1C	10C	1C	10C	1C
**1**	12	17	37	56	28	17
**0.1**	5.7	4.3	17	16	12	16
**0.01**	2.6	2.2	1.7	1.7	4.4	4.0

## Data Availability

The data presented in this study are available on request from the corresponding author.

## Supplementary Materials

The following are available online at https://www.mdpi.com/2079-4991/11/1/197/s1, Table S1: Summary of the attractive and repulsive interactions based on the partial positive or negative charge of substituent groups in molecules interacting with clay; Figure S1: Scheme of conjugation; Figure S1: UV–vis and Fluorescent spectra of all dyes tested at the same concentration in water (gray lines) or 1C of MMT (colored lines); Figure S2: Effect of the MMT (dark/light blue lines) in the red-shift of the absorbance peak; Figure S3: UV–vis and Fluorescent spectra of all conjugated dyes tested at the same concentration in water (gray lines) or 1C of MMT (colored lines); Table S2: E.F. calculated for each nonconjugated and conjugated dye using MMT at different concentrations (10C, 1C and 0.1C); Figure S4: Study of the phenomenon involved in the optical changes of FITC, Chi_FITC, E and Rho; Figure S5: Study of the phenomenon involved in the optical changes of CNF; Figure S6: pH responsiveness of chitosan-conjugated FITC used at different concentrations (1, 0.1 and 0.01 μM) in water, with 10C of MMT and 0.1C of MMT; Figure S7: (A) Scheme of the “off–on” detection mechanism of amino acids using the nonfluorescent fluorescamine. (B) enhancement factor (E.F.) calculated. Figure S9: Characterization of pristine MMT.
