# Supplementary material for: Changes in Optical Properties upon Dye–Clay Interaction: Experimental Evaluation and Applications
Source: Nanomaterials (Basel). 2021 Jan 14;11(1):197. doi: 10.3390/nano11010197 (PMC7830015; doi:10.3390/nano11010197)
Supplement: Supplementary file 1 [file nanomaterials-11-00197-s001.pdf]

Supporting Information

Changes in Optical Properties upon Dye-Clay Interaction: Experimental Evaluation and Applications.

Giorgia Giovannini[1], René M. Rossi[1] and Luciano F. Boesel[1*]

[1] Empa, Swiss Federal Laboratories for Materials Science and Technology, Laboratory for Biomimetic Membranes and Textiles, Lerchenfeldstrasse 5, CH-9014, St.Gallen, Switzerland
[*] Corresponding author

Giorgia Giovannini (giorgia.giovannini@empa.ch), René M. Rossi (rene.rossi@empa.ch), Luciano F. Boesel (luciano.boesel@empa.ch)

Table S1. Summary of the attractive and repulsive interactions based on the partial positive or negative charge of substituent groups in molecules interacting with clay[1].

	Attractive interactions	Repulsive interactions
C	C=O of COOH groups	C in alkane chains<br>C in aromatic rings<br>C in ester groups (COO-R)<br>C in primary and secondary amine (C-NH_2 and C-NH-R)
H	H in alkane chain<br>H in the aromatic ring<br>(they vanish the repulsive effect of C in both cases)	
O		O in ester group (COO-R)
N		N in primary and secondary amine (C-NH_2 and C-NH-R)

To evaluate the possibility of improving dye adsorption on the MMT and the stability of the molecules here studied, FITC, Rho, E, CCF, CNF and CNH_2 were conjugated with chitosan exploiting the presence of primary amino groups in its structure (Figure S1).

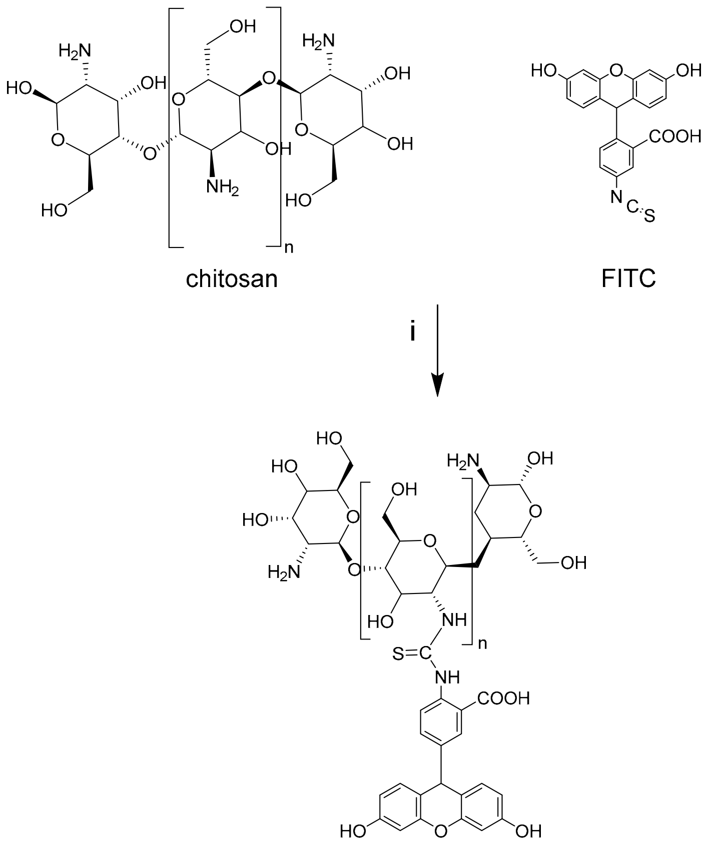

Chi_FITC

Figure S1. Scheme of conjugation. i: Chitosan deacetylated (5 mg) in HCl 1 M (1 mL) stirred for overnight, room-temperature (RT); filtered and neutralized to 6 pH with NaOH; FITC is added reaching 1.1 mM as final concentration (final volume 5 mL). The reaction is stirred for 24 hours, RT; the excess of FITC is removed by dialysis (24h) and centrifugation 8000 rpm, 10 min (x3).

Free dyes (Figure S2, Figure S3) and chitosan-conjugated dyes (Figure S4) were treated with 1C and after 15 minutes absorbance and fluorescent spectra were recorded. The spectra of the dye interacting with MMT were compared with the one measured for the dye in water.

Only relatively small variations were observed in the absorbance spectra of free dyes (Figure S2). In particular, when treated with MMT, FITC and Fluo, the spectra showed an increase in the main peak at 490 nm and a decrease in the intensity for the shoulder at 450 nm which is commonly observed for xanthene dyes and is due to the formation of dimers/aggregates between single molecules[2]. In the case of Rho, the main absorbance peak was slightly red-shifted from 550 nm to 570 nm. The fluorescence enhancement observed when excited at 490 nm was remarkable, almost 5 times higher for FITC and Fluo, while the fluorescent enhancement observed for Rho was less outstanding (intensity increased from 15 to 25 a.u.). In the case of E, only the intensity of the absorbance peak at 520 nm increased from 0.4 to 0.9 a.u. while negligible changes in the fluorescent signal at 544 nm were observed. The opposite results were achieved for CCF in which the absorbance peak was comparable between the water and 1C samples at 400 nm, whereas the fluorescent signal measured at 425 nm in the presence of 1C was 2.5 times higher than in water. When treated with MMT, a bathochromic shift was observed for CNH_2 in the absorbance spectrum (from 340 nm to 360 nm), while the fluorescent signal measured at 450 nm was found to be around 3 times higher than the one measured for CNH_2 in water.

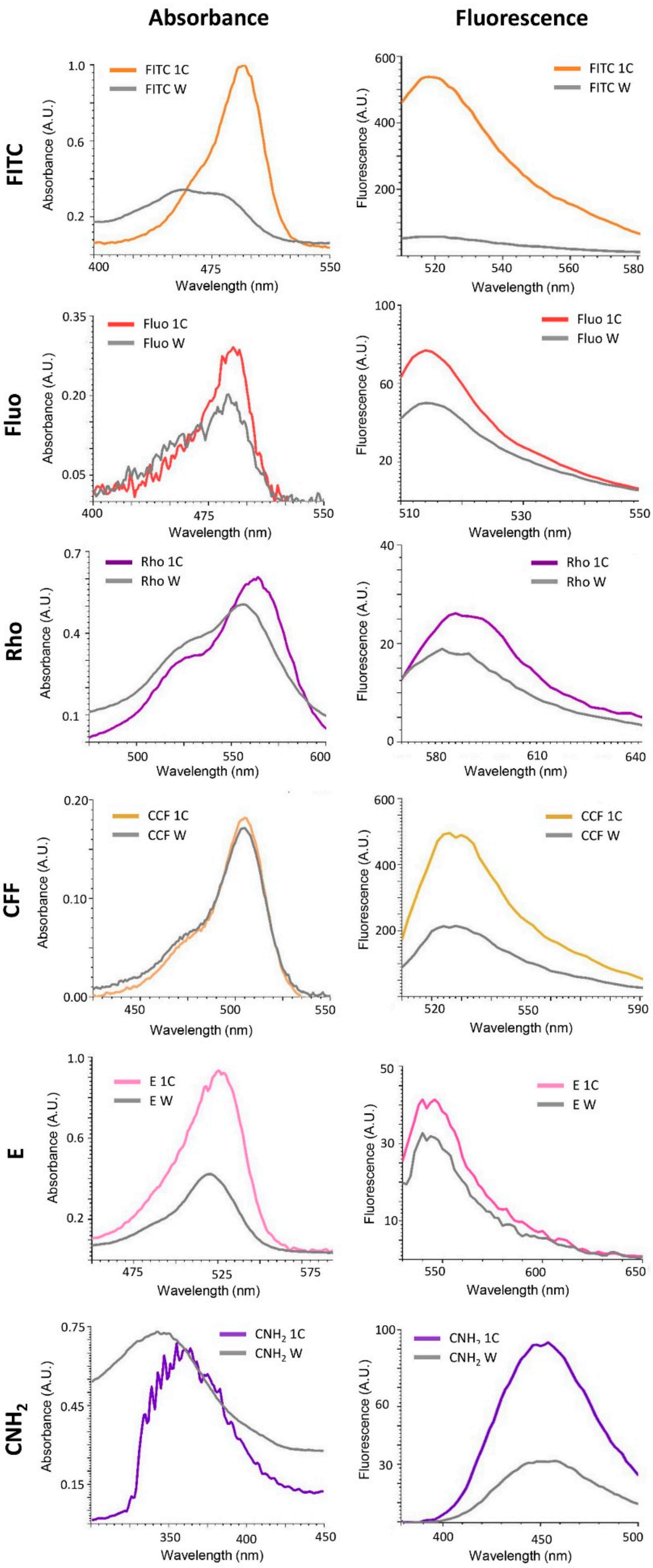

Figure S2. UV-Vis and Fluorescent spectra of all dyes tested at the same concentration in water (grey lines) or 1C of MMT (colored lines). All spectra were normalized to the signals of the corresponding reference (W or 1C suspension). Fluorescent spectra were acquired using the following λex wavelengths: FITC/Fluo 490 nm; Rho 550 nm; CCF 500 nm; E 520 nm; CNH_2 340 nm.

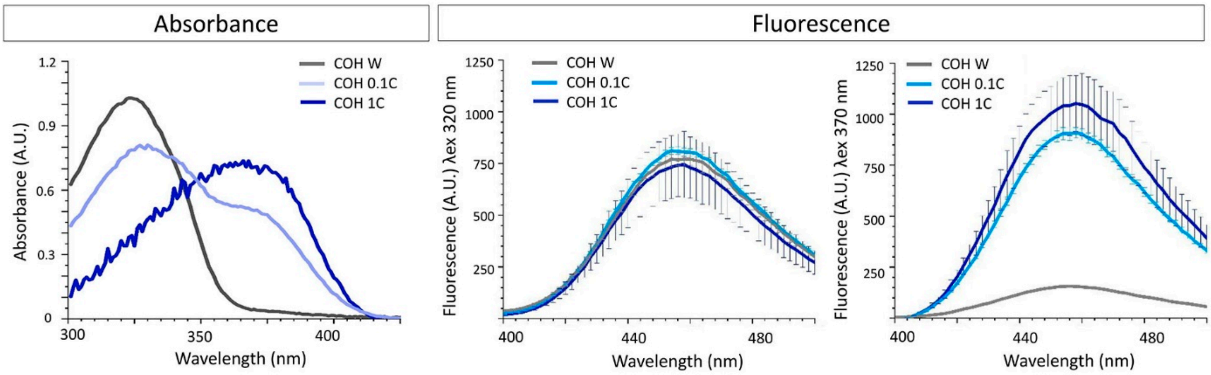

Figure S3. Effect of the MMT (dark/light blue lines) in the red shift of the absorbance peak. All spectra were normalized to the signals of the corresponding reference (W or MMT suspension).

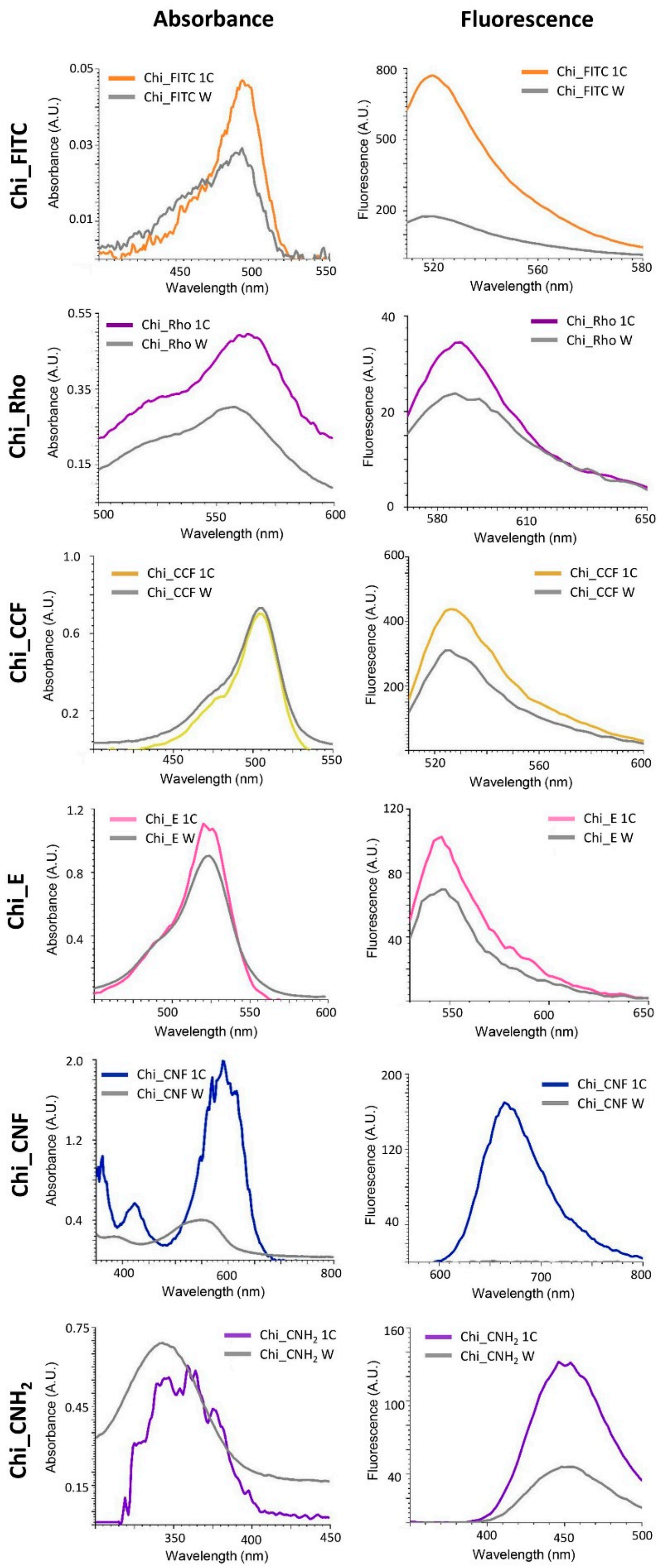

Figure S4. UV-Vis and Fluorescent spectra of all conjugated dyes tested at the same concentration in water (grey lines) or 1C of MMT (colored lines). All spectra were normalized to the signals of the corresponding reference (W or 1C suspension). Fluorescent spectra were acquired using the following λex wavelengths: Chi_FITC 490 nm; Chi_Rho 550 nm; Chi_CCF 500 nm; Chi_E 520 nm; Chi_CNF 550 nm; Chi_CNH$_2$ 340 nm.

The enhancement of the fluorescent signal measured for the non-conjugated and chitosan-conjugated dye was calculated as the ratio between the intensity of the fluorescent signal in the presence of MMT at different concentrations and the intensity measured in water. The values of the so calculated E.F. factor are reported in Table S2. The intensity was determined using the following wavelengths as λ_{ex}-λ_{em} respectively: FITC/Chi_FITC 490-520 nm; Rho/Chi_Rho 550-590 nm; CCF/Chi_CCF 500/525 nm; E/Chi_E 520-550 nm; CNH_2/Chi_CNH_2 340-450 nm.

Table S2. E.F. calculated for each non-conjugated and conjugated dye using MMT at different concentrations (10C, 1C and 0.1C). Values are reported as average values of three independent experiments ± standard deviation.

		non-conjugated		chitosan-conjugated	
		**Average**	**SD**	**Average**	**SD**
**FITC**	**10C**	18	1.6	6.5	0.5
	**1C**	9.6	1.8	4.3	0.4
	**0.1C**	5.3	1.3	2.3	0.2
**Rho**	**10C**	6.0	0.2	6.8	1.2
	**1C**	1.5	0.7	1.4	0.2
	**0.1C**	0.2	0.4	0.1	0.0
**E**	**10C**	2.4	0.3	2.5	0.2
	**1C**	1.2	0.0	1.4	0.1
	**0.1C**	1.0	0.1	1.2	0.1
**CFF**	**10C**	4.3	0.4	3.0	0.1
	**1C**	2.2	0.2	1.4	0.0
	**0.1C**	1.8	0.1	1.2	0.1
**CNH_2**	**10C**	4.1	0.2	3.2	0.5
	**1C**	3.0	0.2	2.9	0.3
	**0.1C**	1.5	0.0	2.5	0.2

Evaluation of the mechanism involved in the optical changes induced by MMT particles on the studied dyes (Figure S5 and S6).

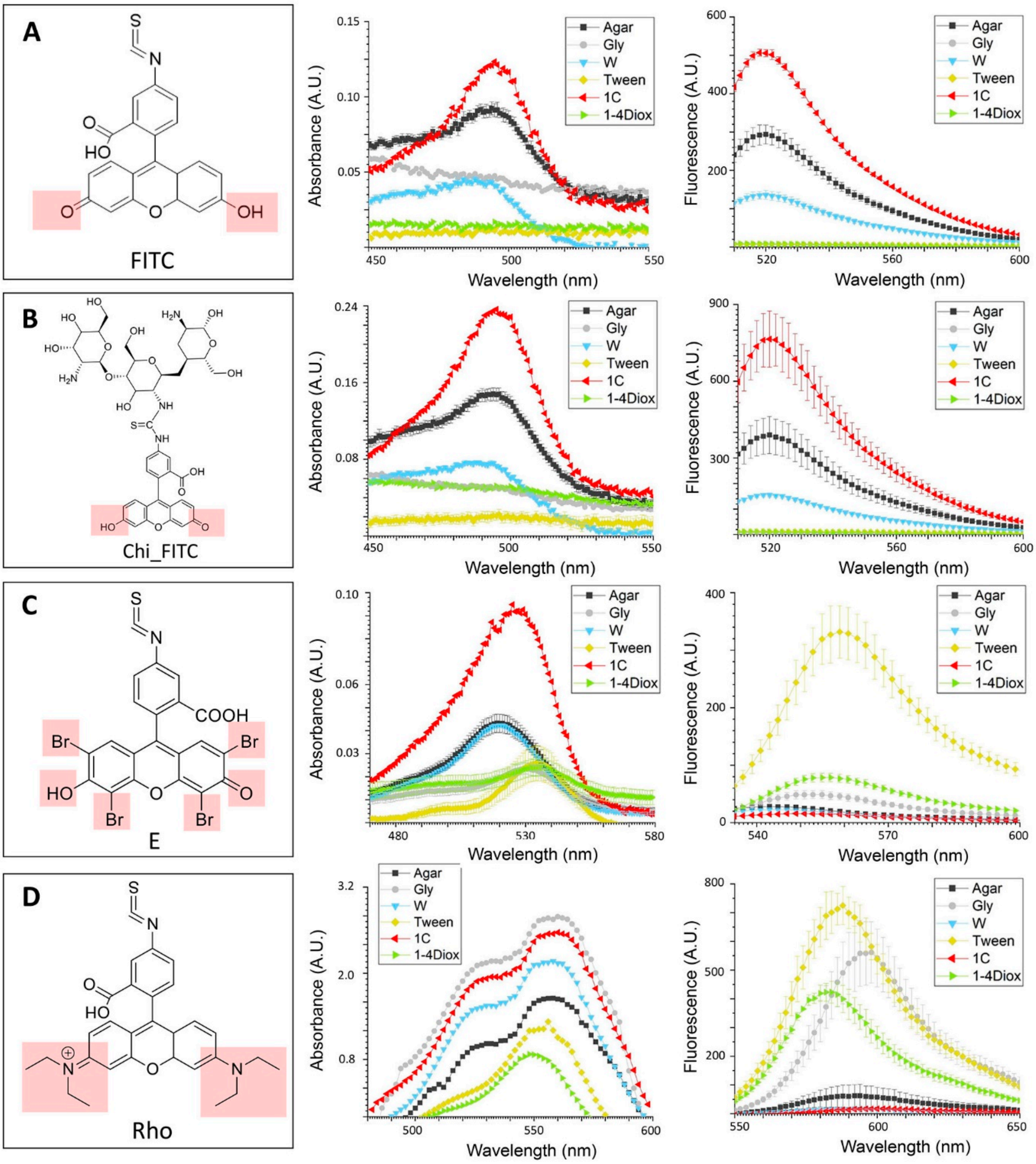

Figure S5. Study of the phenomenon involved in the optical changes of FITC, Chi_FITC, E and Rho. Absorbance and fluorescent spectra are reported for each dye dispersed in different solvents: Agar – black line (hydrogel with high stiffness); Gly - gray line (1.4 Pa·s; 3 OH groups/molecules; propane-1,2,3-triol); Tween - yellow line (0.3 Pa·s; branched polymer with 3 OH group at the end of polyethylene chains; polyoxyethylene sorbitan monolaurate) MMT - red line; 1-4diox – green line (apolar solvent) and water - gray line. All spectra were normalized to the signals of the corresponding solvent and MMT suspension. Fluorescent spectra were acquired using the following λex wavelengths: FITC/Chi_FITC 490 nm; E 520 nm; Rho 550 nm.

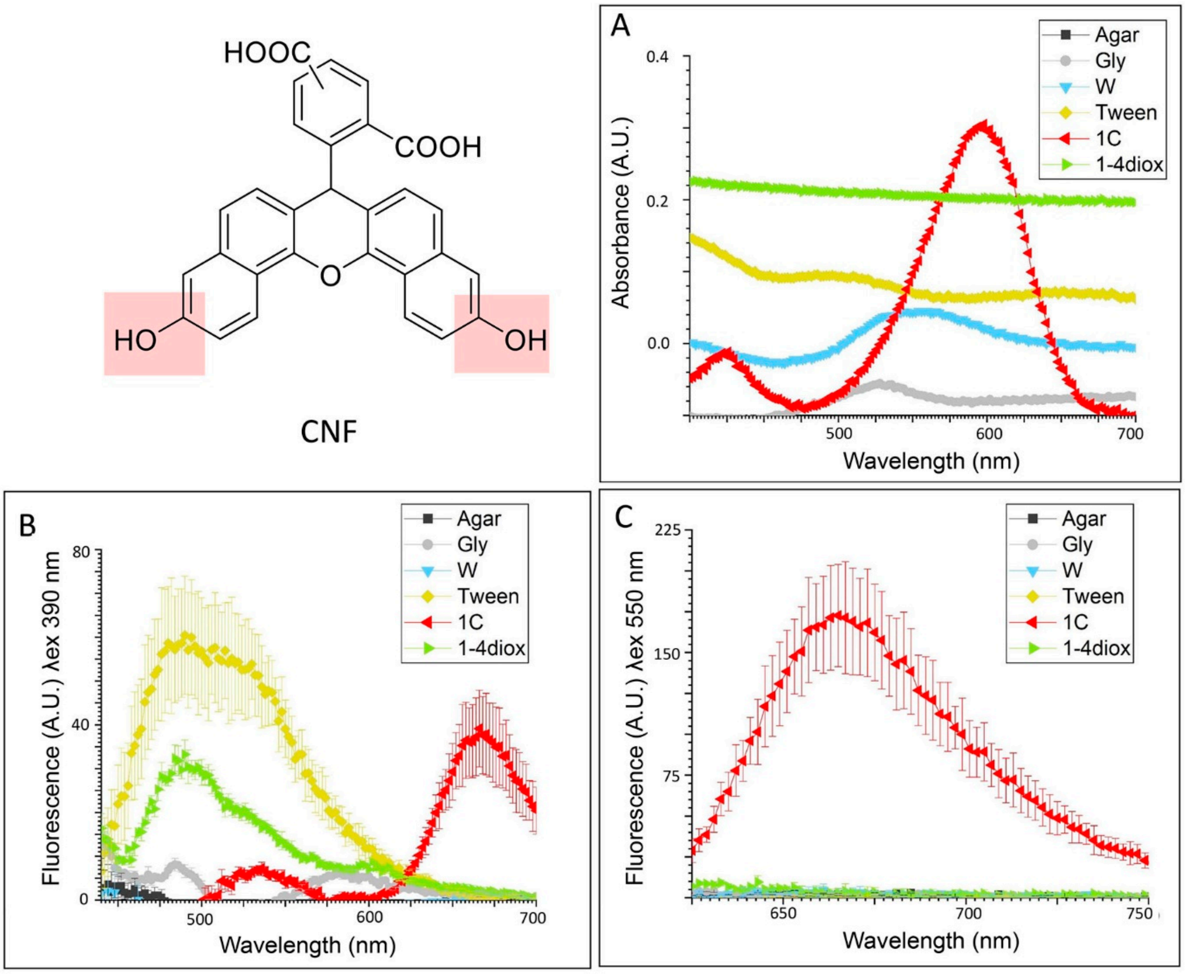

Figure S6. Study of the phenomenon involved in the optical changes of CNF. Absorbance and fluorescent spectra are reported for each dye dispersed in different solvents: Agar – black line (hydrogel with high stiffness); Gly - gray line (1.4 Pa·s; 3 OH groups/molecule; propane-1,2,3-triol); Tween - yellow line (0.3 Pa·s; branched polymer with 3 OH group at the end of polyethylene chains; polyoxyethylene sorbitan monolaurate) MMT - red line; 1-4diox – green line (apolar solvent) and water - gray line. All spectra were normalized to the signals of the corresponding solvent and MMT suspension.

Examples of application of hybrid materials composed of dyes and MMT particles.

Once absorbed on the MMT surface, the pH responsiveness of Chi_FITC was lost. This could be of interest in designing materials in which optical behavior should not be altered by pH variation (Figure S7).

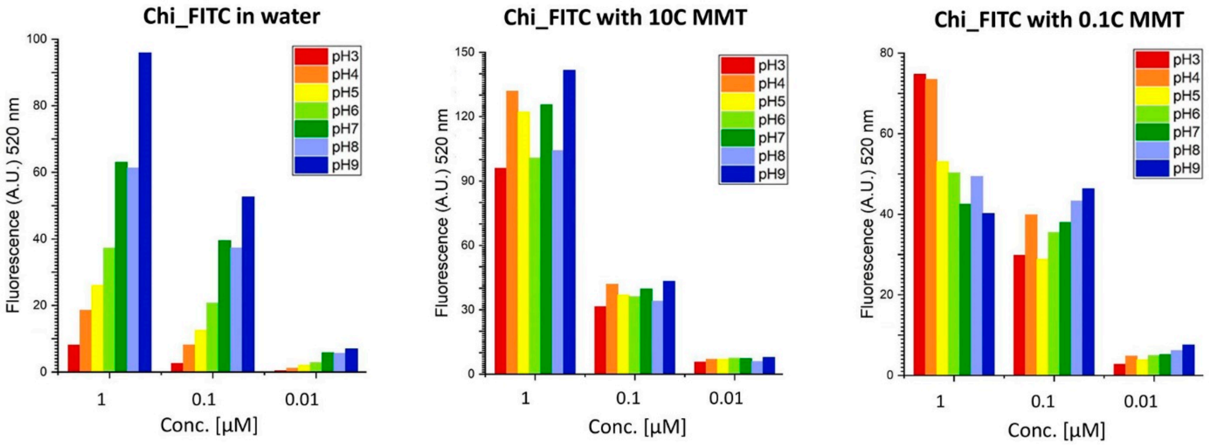

Figure S7. pH responsiveness of chitosan-conjugated FITC used at different concentrations (1, 0.1 and 0.01 μM) in water, with 10C of MMT and 0.1C of MMT. In water the fluorescence intensity of Chi_FITC measured after excitation at 490 nm increases with the increase in pH, in presence of MMT the pH responsiveness of the dye is lost at any concentration tested.

The E.F. achieved using MMT could be exploited to improve sensitivity, decrease the limit of detection (LOD) and reduce the background noise of fluorescence-based detection systems. As proof of concept, we proved the E.F. which can be achieved with fluorescamine, a compound commonly used for the fluorescent detection of amino acids (Figure S8).

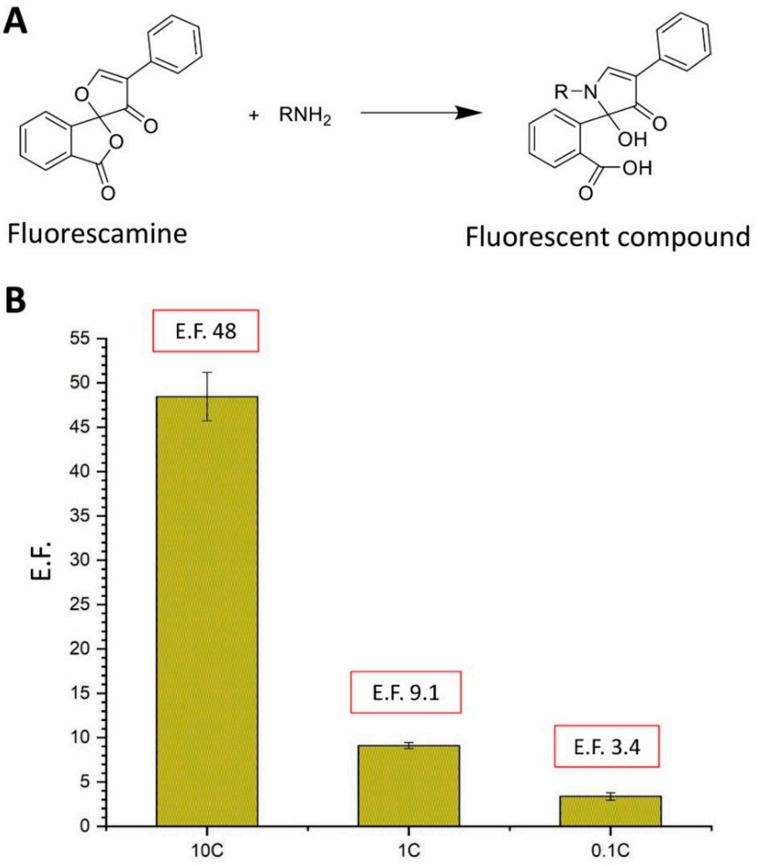

Figure S8. A) Scheme of the 'off-on' detection mechanism of amino acids using the non-fluorescent fluorescamine. B) enhancement factor (E.F.) calculated adding to the fluorescent derivative of fluorescamine after absorption on MMT added at different concentrations (i.e. 10%, 1% and 0.1% wt namely 10C, 1C and 0.1C). The E.F. was calculated as the ratio between the fluorescence intensity measured in water and with MMT. The fluorescent derivative was achieved by reacting fluorescamine with chitosan having several amino groups on the chain.

MMT Characterization:

Scanning electron microscopy (SEM) images were acquired using a Hitachi S-4800 scanning electron microscope at an acceleration voltage of 20 kV. The samples were fixed on a conductive carbon tape and sputter-coated with 5 nm of Au/Pd alloy to facilitate imaging by compensating extensive charging.

X-ray diffraction (XRD) was performed in a Bruker AXS D8 Advance apparatus, using Co Kα radiation (1.79 nm). The conditions used for all specimens were: scanning between 1.9° and 30° (2θ), with a step size of 0.03° (2θ) and 10 s/step. Powder samples were analyzed with the texture technique in order to improve the intensity of 00l reflections of clay layers. Talc (with two main reflections at 11.0° and 21.9°) was used as an internal standard to adjust the position of the reflections of all specimens.

Energy-dispersive X-ray (EDX) analysis was performed using the above mentioned SEM equipped with an INCA X-Sight detector (Oxford Instruments). Three independent specimens were measured and an average value was obtained from the resulting elemental compositions.

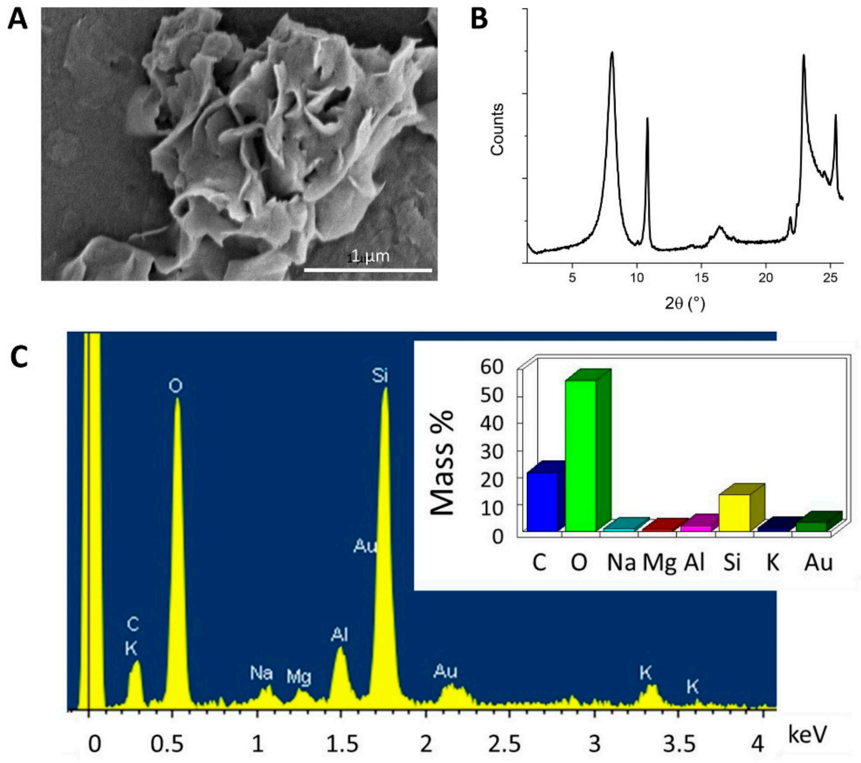

Figure S9. Characterization of pristine MMT. (A) SEM micrograph showing the morphology of the MMT; (B) XRD spectrum of MMT. The peaks at 2θ = 11° and 21.9° are from talc, used as a reference. d_{001} and d_{002} of the pristine clay (8.2°, 1.28 nm and 16.6°, 0.62 nm respectively) are visible. (C) EDX elemental analysis of pristine MMT.

References

1. Katti, D.R.; Katti, K.S.; Raviprasad, M.; Gu, C. Role of Polymer Interactions with Clays and Modifiers on Nanomechanical Properties and Crystallinity in Polymer Clay Nanocomposites. *J. Nanomater.* **2012**, *2012*, 341056, doi:10.1155/2012/341056.
2. Razmkhah, K.; Little, H.; Sandhu, S.; Dafforn, T.R.; Rodger, A. Optical properties of xanthene based fluorescent dyes studied by stretched-film linear dichroism. *RSC Adv.* **2014**, *4*, 37510–37515, doi:10.1039/C4RA06126H.
